# Fun and Balanced Learning Experience: Students’ Perceptions of Serious Games in Higher Education

**DOI:** 10.3390/bs16101766

**Published:** 2026-09-28

**Authors:** Alba Merino-Cajaraville, Salvador Reyes-de-Cózar

**Affiliations:** 1 Department of Communication and Arts, Universidad Loyola, Avda de las Universidades 2, 41704 Dos Hermanas, Sevilla, Spain; 2 Department of Education, Universidad Loyola, Avda de las Universidades 2, 41704 Dos Hermanas, Sevilla, Spain; sreyes@uloyola.es

**Keywords:** serious games, perceived learning, motivation, educational video games, higher education

## Abstract

Serious games offer a pedagogical alternative to mitigate the generational disconnect and demotivation in higher education. However, empirical research often evaluates single game interventions, leaving a gap in understanding general student perceptions rather than learning effectiveness. This study addresses this gap by evaluating the general perceptions of 1302 undergraduate students from 12 Spanish and Dutch universities regarding the pedagogical dimension of serious games based on their prior experiences using the validated Fun And Balanced Learning Experience Scale (FABLE-S). Findings show a positive perception of serious games, with students highly valuing interactivity of the contents, progressive difficulty and goal clarity, while remaining more critical of curriculum alignment and topic organization. Furthermore, statistically significant composite-level differences emerged based on country, gender, area of knowledge, and frequency of play, although these group differences exhibited small effect sizes. A strong positive relationship was also found between perceived learning and motivation. In conclusion, this study positions serious games as positively perceived pedagogical tools based on student perceptions.

## 1. Introduction

Higher education is often described in the literature as being at a turning point, with several authors pointing to a disconnect between traditional pedagogical methods and the profile of new digital learners (Coolsaet, 2024). In this era, characterized by abundant and networked knowledge (Bereznoy et al., 2021), Generation Z’s ability to navigate interactive ecosystems has been argued to sit uneasily with pedagogical structures anchored in sustained attention and unidirectionality (Bradbury, 2016; Philip & Bennett, 2021). Several researchers suggest that, as a result, young people show less tolerance toward these rigid formats and reject methods that ignore their need for active participation (Androshchuk et al., 2022; Bradbury, 2016; Lefterova & Lefterov, 2022; UNESCO, 2023).

This scenario has led to an educational gap whose consequences are manifested in high rates of demotivation and declining student engagement (Pishghadam et al., 2021; Ren & Abhakorn, 2022; Reyes-de-Cózar et al., 2023; Wang & Guan, 2020), a decrease in academic performance (Gamboa-Suárez et al., 2024; Hussein & Abdirahman, 2024), and an increase in dropout rates (Eurostat, 2024).

Therefore, to reconnect with students, the literature points to the need for a transition toward a flexible, collaborative, and learner-centered model capable of offering adaptive environments that respect their own learning paces and preferences. One approach proposed in this regard is active learning. This approach inverts traditional logic by placing the student at the center of their own learning process (Dai & Zhang, 2021; Doolittle et al., 2023; Konopka et al., 2015), promoting participation through practical activities that foster deep and lasting learning (Choi et al., 2021). Within this context, understanding students’ firsthand perceptions becomes paramount. Capturing their subjective experience early on serves as an indicator of pedagogical alignment, determining whether an innovative tool resonates with the end-user or risks being rejected as another format.

Active learning materializes in various strategies, among which game-based learning stands out. This methodology uses play as the core of the educational experience (Byusa et al., 2022), where learning emerges from interaction with ludic environments designed for pedagogical purposes that integrate academic content into their mechanics and narrative (Plass et al., 2015; Roedavan et al., 2021; Schrader, 2022). Within this scenario, serious games emerge as an innovative alternative that projects new possibilities into the classroom (De Freitas & Liarokapis, 2011). Their capacity to connect with current generational narratives, combined with interaction and immersion, offers a resource that moves away from traditional teaching and remains essential to explore in the face of the current educational gap.

Serious games are distinguished by articulating pedagogical objectives within interactive dynamics that foster learning (Bellotti et al., 2011; Tsakarelou et al., 2024). Their advantage lies in how they structure educational practice through active participation, immediate feedback and the adaptation of challenges to student progress (Dunlosky et al., 2013), fundamental principles for the personalization of digital teaching. Thus, their educational contribution stems from a consistent and engaging experience (De Gloria et al., 2014) that facilitates experiential learning through active experimentation (Kolb, 2014).

Extensive literature synthesizes the multifaceted benefits of serious games. Empirical evidence confirms that integrating robust game mechanics with instructional design fosters intrinsic motivation, self-regulation, and problem-solving skills (Huber et al., 2024; López-Serrano et al., 2025; Pacheco-Velazquez et al., 2024), while also promoting long-term knowledge transfer and conceptual understanding (Boyle et al., 2016; Braad et al., 2020; Gee, 2003; Wouters et al., 2013). Furthermore, elements like immersive virtual realities and well-integrated narratives enhance cognitive retention and engagement by providing realistic contexts that make learning meaningful (Alrehaili & Al Osman, 2022; Bush et al., 2023; Naul & Liu, 2020; Rachmatullah et al., 2021).

However, this expanding body of evidence has not been matched by an equivalent theoretical and methodological consolidation, which has simultaneously brought two critical challenges: on one hand, consolidating integrative models in the face of a notable diversification of conceptual frameworks and instruments that point in different directions; on the other, systematically incorporating the student’s perspective into measurement instruments as an active agent of the process.

Regarding the first challenge, it is a priority to find models that advocate for a unified ludic-pedagogical approach, combining both the educational and entertainment components within the same theoretical framework (Haoran et al., 2019; Schrader, 2022; Serrano-Laguna et al., 2018), in order to guarantee the validity of this tool in educational contexts. Currently, scientific literature presents a diversity of conceptual frameworks that can be divided into three main approaches depending on their applicability: prescriptive models for game design, which guide the design of the game itself (e.g., MOTENS, LM-GM); analytical tools for evaluation (e.g., MDA, RETAIN), and reflective frameworks for teacher training (e.g., Four-Dimensional Framework) (Arnab et al., 2015; De Freitas & Jarvis, 2006; Gunter et al., 2008; Hart et al., 2021; Hunicke et al., 2004). The comparison among these frameworks suggests that there is no single model, but rather a set of complementary tools at the disposal of designers, educators, and researchers. This scenario of dispersion calls for an approach capable of systematizing these perspectives into a single, integrative model that can merge both the ludic and pedagogical experience together.

On the other hand, the second challenge to address is reversing the scarce inclusion of the students’ perspective in research, as they are the end-user of the experience (Backlund et al., 2017). Traditional research frequently treats students as passive subjects of intervention rather than active agents, prioritizing technical features or objective performance metrics over the user’s subjective experience (Backlund et al., 2017; Kapralos et al., 2011). Engaging critically with student subjectivity is essential, as the educational utility of a serious game heavily depends on how it aligns with individual preferences and motivational profiles (All et al., 2014; Klock et al., 2020), an alignment that current instruments rarely capture in a systematic way.

Facing both challenges, the FABLE model (Reyes-de-Cózar & Merino-Cajaraville, 2025) emerges as a response to this demand, offering a theoretical base that integrates both perspectives into a single framework. Its structure across seven dimensions provides a solid foundation for developing a measurement instrument capable of capturing the dual and complex nature of serious games: four ludic (game structure, narrative, aesthetics and interaction) and three pedagogical (pedagogical content, educational objectives, and learning process). These dimensions do not operate in isolation but are grounded in established learning theories. The pedagogical core of the model incorporates Piaget’s constructivism (Piaget, 1977) and Kolb’s experiential learning theory (Kolb, 2014), wherein the game’s structural mechanics and interactive aspect of the game serve as environments for active assimilation, accommodation, and immediate feedback loops. This experiential nature of the game directly drives cognitive engagement, as the clarity of objectives and progressive scaffolding prevent cognitive overload while sustaining deep concentration. Simultaneously, this cognitive flow is intrinsically linked to the affective domain via Self-Determination Theory (Ryan & Deci, 2017). The ludic dimensions of the framework (game structure, narrative, interaction and aesthetics) foster a sense of autonomy and competence during gameplay. Ludic experience sustains the psychological needs required for motivation, which in turn fuels the cognitive engagement necessary for constructive and meaningful learning.

Building on this integrative structure, Merino-Cajaraville and Reyes-de-Cózar (2026) propose the FABLE-S (Fun And Balanced Learning Experience-Scale), an instrument that operationalizes the FABLE model while placing students’ own perceptions at the center of its measurement approach. By gauging their perceptions, the scale establishes itself as a reliable and valid tool that evaluates game design as well as the student’s subjective experience with the serious game. Likewise, most empirical research on educational gamification restricts its evaluation to specific interventions using a single serious game. This study adopts a complementary approach, evaluating higher education students’ general perceptions of serious games based on their prior experiences rather than a single intervention, in order to capture a broader perspective of learner perceptions across the range of games students have encountered. Measuring these general perceptions alongside the FABLE-Scale allows for a comprehensive mapping of how students perceive educational dimension of serious games. To complement these educational dimensions, this study incorporates two independent, single-item indicators: perceived learning and motivation. While the scale’s pedagogical dimensions (content and objectives and learning process) evaluate the specific instructional mechanisms of serious games, these single items capture the students’ self-reported perceptions of their cumulative experience on these variables.

Based on the premises above, this study aims to address the need to understand the student’s perception firsthand and proposes to evaluate the educational dimension of serious games by placing the student’s perception at the center of the analysis. It should be noted that this study does not test whether serious games are effective in reversing generational disengagement from traditional teaching methods; rather, it examines students’ perceptions of the educational dimensions of serious games as an initial, exploratory indicator of their potential pedagogical alignment with this student profile. To that end, this research addresses the following objectives:

- O1: To assess the evaluation that higher education students assign to the educational dimensions of serious games;
- O2: To examine whether there are significant differences in students’ perceptions based on sociodemographic and attitudinal variables;
- O3: To evaluate, through an independent global indicator, students’ overall perception of the learning derived retrospectively from their experience with serious games;
- O4: To explore, through an independent global indicator, the overall level of motivation students perceive when playing serious games;
- O5: To determine whether relationships can be established between the educational dimensions of serious games, the perceived level of learning, motivation and frequency of play.

The remainder of this paper is structured as follows. Section 2 describes the methodology, including research design, sample, instrument, and data collection procedure. Section 3 presents the descriptive, inferential, and correlational results. Section 4 discusses empirical findings considering previous literature and details the study’s limitations and future research lines. Finally, Section 5 draws the main conclusions of the research.

## 2. Materials and Methods

### 2.1. Research Design

This research employs a quantitative, cross-sectional, non-experimental survey design, using a descriptive, comparative, and correlational approach. This design was selected to capture students’ general perceptions of serious games based on their prior experience, rather than to evaluate the effect of a specific intervention, and to examine associations without establishing causality between the study variables. The research respects participants’ natural gaming habits during data collection to ensure objectivity and a non-intrusive approach.

The dataset analyzed in the present study is derived from the sample of 1302 higher education students used in the psychometric validation of the Fun And Balanced Learning Experience Scale (FABLE-S) (Merino-Cajaraville & Reyes-de-Cózar, 2026). This research constitutes a secondary, planned analysis of the validation dataset, specifically designed to address a distinct set of objectives that do not overlap with the previous publication. While the validation study focused on establishing the instrument’s psychometric properties (factor structure, reliability, and validity), the present study explores students’ pedagogical perceptions of serious games, examines differences across sociodemographic and attitudinal variables, and tests the correlation between pedagogical dimensions, perceived learning, and motivation. No descriptive statistics, group comparisons, or correlational analyses reported in this manuscript have been published elsewhere.

### 2.2. Sample

The total sample consisted of 1302 higher education students, recruited from 12 public and private universities across Spain (*n* = 830) and the Netherlands (*n* = 472). A detailed breakdown of the sample distribution by country and participating university is provided in Table S1. All the students were enrolled in undergraduate courses (from 1st to 4th year) and represented every area of knowledge (Arts and Humanities, Engineering and Architecture, Social and Human Sciences, Health, and Sciences). Sample distribution is shown below (see Table 1).

### 2.3. Data Collection

The sample for this study was selected using a non-probability convenience sampling method. Fieldwork was conducted between April 2024 and December 2024. The sample was reached through an in-person field study conducted in cooperation with instructors teaching different subjects across various fields of study. These teachers allocated a segment of their lecture time to allow the researchers to visit the classrooms face-to-face. Regarding the sample size, its determination was based on convenience, feasibility, and the maximum availability of courses and students during the data collection period, aiming to maximize the number of participants to ensure a diverse representation across different fields of study. Participants’ natural gaming and educational histories were respected during data collection to ensure objectivity. Because the evaluation is based on students’ general perceptions from prior experiences rather than an intervention with a specific serious game, the sample captures a diverse socio-demographic spectrum across 1302 students.

Data collection was carried out using the Qualtrics platform, and to ensure the accessibility of the study, the instrument was translated and made available in both Spanish and English. Because the instrument was administered in both Spanish (Spain) and English (The Netherlands), a cultural adaptation process was followed. The scale was originally created and validated in Spanish (Merino-Cajaraville & Reyes-de-Cózar, 2026). To develop the English version, two bilingual researchers in educational technology performed a forward translation. Discrepancies were resolved through committee review to maintain semantic, idiomatic, and conceptual equivalence across both language versions before deployment.

To control for potential procedural sources of Common Method Bias (CMB) (Podsakoff et al., 2003), several measures were implemented during data collection: (a) complete participant anonymity was guaranteed to reduce social desirability and evaluation apprehension; (b) clear psychological separation was maintained between the multi-item FABLE-S scale sections and the independent global single-item measures (perceived learning and motivation); and (c) item ambiguity was minimized by providing a standardized definition and concrete higher education examples of serious games prior to questionnaire completion.

In line with the last criterion mentioned, to ensure a shared understanding of the construct before administering the survey, researchers contextualized the concept of serious games for all participants. The following standardized definition was presented on screen: ‘Serious games are digital games designed for educational purposes, where learning is integrated directly into gameplay mechanics.’ This definition was accompanied by concrete, familiar examples, such as MineCraft Education Edition. Subsequently, a screening question was posed to determine whether respondents had prior experience interacting with these tools. Participants who reported no prior experience with serious games were asked to leave the session. Remaining participants were then instructed to complete the questionnaire by reflecting cumulatively on their overall experiences with such educational tools. To control for potential confusion and cognitive bias arising from the scale’s generic singular phrasing (a standard Spanish grammatical construction used to refer to a medium or category in general), researchers supervised data collection and were available to clarify any questions participants raised.

### 2.4. Instrument

To ensure methodological rigor, this study utilizes the validated FABLE-Scale (Merino-Cajaraville & Reyes-de-Cózar, 2026), grounded in the FABLE model (Reyes-de-Cózar & Merino-Cajaraville, 2025) and designed to measure students’ perceptions of serious games and their perceived learning and motivation levels.

The psychometric properties of the FABLE-Scale were established in its original development and validation study (Merino-Cajaraville & Reyes-de-Cózar, 2026). Confirmatory factor analysis (CFA) supported the factor structure with empirical fit indices meeting rigorous thresholds (χ^2^/df = 3.29, CFI = 0.964, TLI = 0.957, RMSEA = 0.042 [90% CI: 0.038, 0.046], SRMR = 0.031). Furthermore, invariance testing supported an equivalent factor structure across the Spanish (CFI = 0.962, RMSEA = 0.045) and Dutch (CFI = 0.936, RMSEA = 0.051) subsample. For the current sample (*N* = 1302), internal consistency estimates were evaluated separately for the two analyzed pedagogical composites, demonstrating high reliability: *Content and Objectives* exhibited Cronbach’s alpha = 0.814 and McDonald’s omega = 0.817, while *Learning Process* yielded Cronbach’s alpha = 0.839 and McDonald’s omega = 0.840.

As detailed in Table 2, the FABLE-Scale is theoretically structured around two macro-dimensions: Ludic Experience (comprising *Game Structure, Narrative, Aesthetics* and *Interaction*) and Pedagogical Experience (comprising *Content and Objectives* and *Learning Process*). While the conceptual FABLE model originally proposes seven theoretical dimensions (four ludic and three pedagogical), the empirical validation of the operational FABLE-S scale demonstrated a six-factor structure, wherein pedagogical content and educational objectives unified into a single composite factor entitled *Content and Objectives* (C&O) alongside the *Learning Process* (PRO) dimension.

Of the seven dimensions shown, this study focuses exclusively on the two dimensions comprising the Pedagogical Experience component: *Content and Objectives* (C&O) and *Learning Process* (PRO). Given that the core objectives of this research are focused on the educational and pedagogical perceptions of serious games, the Pedagogical Experience has been intentionally selected. The remaining dimensions from the Ludic Experience were excluded as they measure technical, narrative, and artistic attributes that fall outside the specific scope and scientific aims of this study. Following the Pedagogical Experience and its corresponding dimensions (*Content and Objectives* and *Learning Process*), the specific items that comprise them utilized in this research are shown in Table 3.

The dimensions selected from FABLE-S comprise 10 items. All items are evaluated on a Likert-type scale from 1 to 5, with 1 being the minimum value (Not at all) and 5 being the maximum value (A lot). In addition to those items, the instrument includes other questions related to sociodemographic variables (country, age, gender, academic year, and area of knowledge) and attitudinal variables (frequency of play). Finally, to measure perceived learning and motivation, students were asked to indicate their perception of the learning experienced through serious games and how motivated they generally feel when engaging with these tools.

### 2.5. Statistical Analysis

Three statistical analyses were performed to reach the objectives of this study.

- Descriptive Statistics. Descriptive statistics, such as frequency, means, and standard deviations, were calculated to identify which educational items were best and worst perceived by students (O1) and to evaluate the levels of perceived learning (O3) and motivation (O4). Descriptive statistics are performed using SPSS, version 24;
- Hypothesis testing. Hypothesis testing (ANOVA) and post hoc tests were conducted to examine whether there were significant differences in students’ perceptions based on country, age, gender^1^, academic year, area of knowledge, or frequency of playing (O2). To test subgroup differences across variables, a hierarchical hypothesis testing strategy was implemented. Inferential statistical testing on the two composite scores (C&O and PRO) was defined as the primary outcome evaluation. Individual item-level comparisons across the 10 scale items were classified as secondary exploratory analyses. To control the accumulation of Type I errors across exploratory tests, the Benjamini–Hochberg (BH) false discovery rate (FDR) correction was applied. Hypothesis testing is performed using JASP, version 0.19.0.1;
- Correlational Analysis. Pearson correlation coefficients (*r*) were calculated alongside non-parametric Spearman rank-order correlations (*rho*) as a sensitivity analysis for ordinal scale assumptions to explore the relationships between educational dimensions, perceived learning and motivation levels, and frequency of play (O5). Correlational analysis is performed using Stata, version SE 18.

### 2.6. Ethical Considerations

This study complies with the Ethical Principles and Code of Conduct of the American Psychological Association (APA), in accordance with the Declaration of Helsinki and has received approval from the Ethics Committee of Loyola University (approval code: 240402 CE24518). Likewise, it should be noted that the questionnaire includes an information sheet and informed consent form at the beginning, which detail the objectives of the research as well as the participants’ rights, ensuring the anonymity of students throughout the entire process. For first-year undergraduate students aged 17, the approved ethical protocol authorized direct informed consent within the university classroom setting, given that participants were matriculated higher education students engaging in an anonymous, voluntary, and minimal-risk survey.

## 3. Results

### 3.1. Students’ Perceptions of Educational Dimensions in Serious Games

A descriptive analysis using frequencies, mean, and standard deviation was carried out to assess the evaluation that higher education students assign to the educational dimensions of serious games, in line with O1. When observing students’ perceptions of the educational dimensions from a general perspective, differences in the responses are observed (see Figure 1).

On the one hand, the items with the highest scores are related to interactivity and learning scaffolding, the gradual difficulty of tasks and the clarity of objectives. 78.42% of the students value quite a lot or a lot that *the learning process is interactive*, as well as 79.26% value with the highest scores that *the new knowledge helps them overcome later levels*. Furthermore, 79.19% of the students report high scores for *the task increase in difficulty progressively*, while 77.88% rate the item *the objectives are clear*.

On the other hand, specific aspects of the content such as its division into learning units, its suitability for learning, and the extent to which the content is relevant to the curriculum are the items rated lowest by higher education students. Specifically, 16.90% of the students place little to no importance on the item *educational content aligns with my curriculum*, while 11.98% express a similar sentiment regarding *educational content is organized into learning units*. Also, 9.06% of the students report low scores for *educational content is appropriate for my learning*.

### 3.2. Descriptive Results for Content and Objectives and Learning Process

The following section analyzes the descriptive statistics to gain insight into students’ perceptions, focusing on the two key educational dimensions: *Content and Objectives* and *Learning Process*.

Focusing on the items of the *Content and Objectives* dimension, the responses reveal a positive assessment by the students (see Table 4).

The clarity of educational objectives is the most valued aspect (*M* = 4.08), and the structure clarity of the contents concentrates the most unanimous responses (σ = 0.94). On the other hand, the content relevance for the curriculum is the aspect with the lowest score (*M* = 3.67) and the highest standard deviation (σ = 1.18).

Regarding the items of the *Learning Process* dimension, students show an overall positive perception (see Table 5).

The item associated with the learning process that students value the most is their interactivity (*M* = 4.12), as opposed to the learning transfer and immersion, which are the items with the lowest means (*M* = 4.03). By contrast, the item related to scaffolding elicited the most unanimous responses among students (σ = 0.88), whereas the item on knowledge transfer showed the greatest variability (σ = 0.98).

To perform a general analysis of the educational dimensions, the composite indices of both *Content and Objectives* and *Learning Process* dimensions have been calculated. These composite indices were calculated as the means of the five items reported in Table 4 and Table 5 for each dimension. This composite-level analysis complements the item-level results by allowing a more parsimonious comparison of the two pedagogical dimensions (see Table 6).

The descriptive analysis reveals that the means for the two evaluated indices of the dimensions (C&O and PRO) are high, indicating a generally positive perception of educational dimensions in serious games.

### 3.3. Differences in Students’ Perceptions by Sociodemographic and Gaming Characteristics

To answer O2, hypothesis testing was conducted to examine whether there were significant differences in students’ perceptions of educational dimensions of serious games based on country, age, gender, academic year, area of knowledge, or frequency of playing.

Prior to the analysis, normality (skewness/kurtosis, Shapiro–Wilk) was assessed for all study variables. Skewness (−0.63 to −1.07) and kurtosis (−0.21 to 2.02) fell within acceptable limits for parametric testing despite significant Shapiro–Wilk results (*p* < 0.001) given the size of the sample (*N* = 1302). Since the assumption of homogeneity of variances (Levene’s test) was not met for several grouping variables, Welch’s ANOVA and Games–Howell post hoc tests were used for all comparisons (Delacre et al., 2017). For Pearson correlations, linearity was confirmed by comparing linear and quadratic model fits (ΔR^2^ < 0.01 for all pairs).

Given the large number of ANOVA tests conducted, the risk of Type I error accumulation should be considered when interpreting item-level results. Findings at the composite (dimension) level are therefore given interpretive priority over isolated item-level significant differences. Accordingly, post hoc comparisons were conducted at the composite index level.

The results are shown below (see Table 7).

Focusing on the *Content and Objectives* dimension, the analysis reveals that while country, age, academic year, area of knowledge, and frequency of play show limited influence on the perception of the educational dimensions of serious games, statistically significant differences are observed based on students’ gender. No statistically significant differences are found across areas of knowledge in the evaluated items, except for item C&O_2, indicating a consistent perception regardless of students’ field of study. The frequency of play shows significant differences for item C&O_1, indicating that this variable plays a role in how students perceive the clarity of the content structure. Finally, gender emerges as the most influential variable in students’ perceptions of the *Content and Objectives* dimension, with significant differences observed across all items except C&O_4, where regardless of gender students more uniformly rate the alignment of the content with the curriculum.

Regarding the *Learning Process* dimension, no significant differences are observed based on age, gender, or academic year. However, the variables of country, area of knowledge, and frequency of play show significant differences, all at the index level and when disaggregated by items. Regarding the country variable, neither PRO_1 nor PRO_4 show significant differences, indicating that students from both Spain and the Netherlands similarly value engagement with learning and the progressive difficulty of the tasks. With respect to the area of knowledge, the only item that does not show significant differences is PRO_4, which implies that engagement with learning is consistent across areas of knowledge.

For variables showing statistically significant differences at index level, a Games–Howell post hoc test was conducted (see Table 8) to compare group means after conducting the ANOVA test. This approach enables multiple pairwise comparisons to identify which groups differ significantly.

Regarding *Content and Objectives* dimension, significant differences are observed by gender (*p* < 0.001). Games–Howell post hoc tests confirm that women (*M* = 3.99) assign a significantly higher valuation to this dimension compared to men (*M* = 3.76). Nonetheless, the effect size for this difference is small (*d* = 0.295), suggesting that while mathematically robust, its practical significance in classroom settings may be limited.

Regarding the *Learning Process* dimension, analyses reveal statistically significant differences across three of the evaluated variables: country, area of knowledge, and frequency of play. First, a significant difference is found based on the students’ country of study (*p* = 0.035). The Games–Howell post hoc test indicates that students from Spain attain a significantly higher valuation (*M* = 4.12) in this dimension compared to those from the Netherlands (*M* = 4.00), although the effect size for this difference is small (*d* = 0.167), denoting restricted practical significance. Secondly, significant differences are observed regarding the area of knowledge (*p* = 0.008). Pairwise comparisons reveal that Social Sciences students report lower scores (*M* = 3.96) than students from other fields, such as Engineering and Architecture (*M* = 4.15), Arts and Humanities (*M* = 4.23), and Health (*M* = 4.14). In this case, the contrast with the largest, though still small, effect size is between Social Sciences and Arts and Humanities (*d* = 0.372). Finally, significant differences emerge when analyzing students’ perceptions according to their frequency of play (*p* < 0.001). Post hoc contrasts demonstrate that daily players report significantly higher valuations (*M* = 4.34) than those who never play (*M* = 3.92) or almost never play (*M* = 4.02), play occasionally (*M* = 4.13) or frequently (*M* = 4.14). Notably, the contrast between the daily and never pair yields a moderate effect size (*d* = 0.592), representing the most robust effect observed in this dimension.

### 3.4. Students’ Assessment of the Level of Perceived Learning and Motivation

To address O3 and O4, students were asked to assess how much they feel they learn and to rate the extent to which they feel motivated when playing a serious game in order to evaluate the levels of perceived learning and motivation (see Figure 2).

The findings indicate that responses predominantly fall within the higher values of the scale in both cases, both for perceived learning (*M* = 3.79) and for motivation (*M* = 3.99). The data show that the highest levels (4 and 5) concentrate most of the responses, with 72.8% in motivation and 63.9% in perceived learning. The lowest levels (1 and 2) present reduced percentages, with 11.4% in perceived learning and 8.2% in motivation.

### 3.5. Correlation Between Perceived Level of Learning, Motivation, and Frequency of Play

In line with O5, the Pearson correlation coefficient is used to determine the possible significant relations between educational dimensions elements, perceived learning, motivation and frequency of play, and the extent to which they are significant. The results obtained are shown below (see Table 9).

First, regarding *Perceived learning*, the data shows a positive and statistically significant correlation with both *Content and Objectives* (*r* = 0.484, *p* < 0.001; *rho* = 0.453, *p* < 0.001) and the *Learning Process* dimensions (*r* = 0.477, *p* < 0.001; *rho* = 0.457, *p* < 0.001). Both correlations represent moderate associations with moderate practical significance.

Second, *Motivation* reveals positive and statistically significant correlations across all analyzed dimensions. Specifically, it presents its strongest correlation with *Perceived learning* (*r* = 0.589, *p* < 0.001; *rho* = 0.596, *p* < 0.001).

Third, regarding *Frequency of play*, despite presenting statistically significant correlations with the *Learning Process* dimension (*r* = 0.070, *p* = 0.012; *rho* = 0.064, *p* = 0.020) and *Motivation* (*r* = 0.156, *p* < 0.001; *rho* = 0.123, *p* < 0.001), all observed coefficients are low. From a statistical standpoint, these weak values indicate that the relationship between students’ gaming habits and their scores on these dimensions is minimal.

## 4. Discussion

The empirical findings of this study offer essential insights into how higher education students, as active end-users, experience and perceive the play and pedagogy within serious games. By placing the learner’s firsthand perception at the center of the analysis through the FABLE-S scale, this research addresses the broad generational challenge of demotivation by empirically studying serious games, giving voice to students as active agents of the process. From the results, some findings emerge: the perception of the pedagogical dimensions, *Content and Objectives* (C&O), and *Learning Process* (PRO), are conditioned by attitudinal variables. Consequently, the following discussion interprets these differentiated responses to understand how student profiles shape the success of serious games in contemporary higher education. As this study captures undergraduates’ subjective perceptions rather than direct learning outcomes, these findings reflect the psychological and pedagogical readiness of university students rather than objective serious games efficacy.

### 4.1. Main Results

#### 4.1.1. Interactivity and Structured Learning as Drivers of Positive Educational Perception

The primary objective of this study was to examine higher education students’ retrospective perceptions of serious games across two pedagogical dimensions: *Content and Objectives* (C&O) and *Learning Process* (PRO). Overall, the descriptive findings indicate a positive perception of serious games as instructional tools. Differentiated responses are observed upon deeper analysis. Focusing on the items with the highest scores, the responses reveal a positive assessment by the students of the interactivity and learning scaffolding, the gradual difficulty of tasks and the clarity of educational objectives, aligning with other studies in the literature. Locke’s Goal-Setting Theory (Locke, 1968) supports students’ preference about clarity and realism of the objectives, arguing that setting specific, challenging, yet realistic goals are more effective for learning. This preference is also backed by the literature, as Wang et al. (2017) point out that serious games with clear, well-defined objectives promote greater concentration and enjoyment during learning, thereby enhancing engagement levels (Froiland & Worrell, 2016; Lane et al., 2021; Sun et al., 2023).

Along the same lines of the most highly rated aspects are interactivity and knowledge scaffolding. Grounded in Piaget’s constructivist perspective (Piaget, 1977), direct interaction with content is often preferred by students as it actively drives the mechanisms of assimilation and accommodation, which are foundational prior to achieving learning transfer. To shift this perspective, authors like Black and Allen (2017) argue that, to achieve greater student recognition of knowledge transfer, it is essential to incorporate real-life situations into the learning experience. Recreating realistic scenarios within an interactive context leads to more motivating and meaningful learning (Dankbaar, 2017).

On the other hand, the lowest-rated items correspond to content-related aspects, such as its division into learning units, its suitability for learning, and the extent to which the content is relevant to the curriculum. This outcome aligns with the empirical findings of Iten and Petko (2016), who demonstrated that high levels of game enjoyment do not automatically translate into positive ratings for instructional value, as students tend to remain highly critical of content suitability and its alignment with formal learning goals. This outcome highlights that while students value the interactivity of serious games, they remain skeptical of its academic rigor (Boyle et al., 2016). Educational games are frequently perceived as supplementary activities rather than integrated curricular tools (De Freitas, 2014; Whitton, 2014). Under the lens of Self-Determination Theory (Ryan & Deci, 2017), if digital content feels disconnected from the core learning process, students detect a lack of curriculum alignment, refusing to accept the game as a legitimate learning platform (Iten & Petko, 2016; Lameras et al., 2017).

These findings carry practical implications for instructional design and discipline-specific pedagogy. Educators and game designers must move away from models where learning content is merely layered on top of play. Instead, the core gameplay mechanics must directly mirror the professional competencies of the curriculum. Furthermore, when introducing serious games in higher education, instructors must provide explicit scaffolding and debriefing sessions to connect the game’s structural tasks with formal learning objectives, ensuring students perceive the experience as a rigorous environment.

#### 4.1.2. Effects of Sociodemographic and Attitudinal Variables in the Evaluation of the Educational Dimension of Serious Games

When examining subgroup differences across composite scale scores, statistically significant variations were identified, though most group differences exhibited small effect sizes. The comparative analysis revealed statistically significant differences based on country, gender, area of knowledge, and frequency of play, with no differences by age or academic year.

Considering country, this variable shows significant differences within the *Learning Process* dimension, with students from Spain evaluating the items of this dimension more favorably. In accordance with methodological caution, this variation should not be attributed to national culture, as cultural dimensions were not directly measured in this study. Country of study was inherently confounded with specific university institutions, course enrollment, language version, and recruitment procedures. Consequently, this difference is more appropriately understood as reflecting sample-specific and institutional variations across participating universities.

Attending to gender, the *Content and Objectives* dimension shows significant differences across all its items. This finding suggests that female undergraduates in this sample reported higher appreciation for structured learning goals and explicit curricular alignment in digital tools. This aligns with prior literature indicating that female learners often place high value on clear task organization and instructional guidance (Barria-Díaz et al., 2023).

Regarding area of knowledge, both dimensions show significant differences. In *Content and Objectives*, a more subtle pattern is observed, with differences appearing only at the index level and in a single item (*Content is appropriate for my learning*), where Health students evaluate this dimension more favorably compared to those in Social Sciences and Sciences. In terms of the *Learning Process* dimension, pairwise comparisons revealed that Social Sciences students reported lower scores than students from other fields, such as Engineering and Architecture, Arts and Humanities, and Health. A cross-dimensional consistency emerges regarding the gap between Health (which scores high) and Social Sciences (which scores low), a pattern that repeats across both dimensions.

Concerning frequency of play, differences are observed in the *Learning Process* dimension. In both cases, post hoc pairwise contrasts demonstrated that students who play daily report significantly higher scores than those who never or almost never, or only occasionally play, yielding moderate effect sizes. Rather than personal gaming habits directly conditioning cognitive learning outcomes, this finding indicates that personal familiarity with video game environments co-occurs with more favorable retrospective appraisals of interactive instructional mechanics, in line with literature findings (Hamari et al., 2016).

Although these group differences reached statistical significance, their corresponding effect sizes were predominantly small, indicating that these profile variations should be interpreted with caution and do not imply barriers to implementation.

#### 4.1.3. Correlations Between Perceived Learning, Motivation, and Frequency of Play

When evaluated through independent global indicators, students reported high levels of perceived learning and motivation, though a minority reported low values, highlighting the need to refine implementation for diverse motivational profiles (Sailer & Homner, 2020). This finding aligns with previous research highlighting the ability of serious games to facilitate content acquisition through experimentation and immersion (Qian & Clark, 2016). However, the fact that 1 out of 10 students reported low or no learning indicates that there is a segment whose learning experience does not appear to benefit from this tool. Furthermore, the observed distribution, where approximately one in four students places their learning at intermediate levels, suggests the need to further refine the implementation of serious games in order to maximize their educational impact across different learning profiles (Lameras et al., 2017).

In the same line, the results confirm students perceive this tool as motivational, in line with research in the literature (Ryan & Rigby, 2020). The fact that nearly three-quarters of students report high levels of motivation suggests that serious games are effectively fulfilling one of their central aims: transforming the learning experience into a more engaging and stimulating activity (Plass et al., 2015; Sipiyaruk et al., 2018; Yu et al., 2022). However, the presence of students reporting low motivation warrants consideration. Although lower than in the case of perceived learning, this result may suggest that barriers remain for a portion of the student population. These differences can be taken as hypotheses for future research.

Finally, the strong correlation between perceived learning and motivation constitutes a notable finding indicating that students who positively rate their learning experience also report higher overall motivation when playing serious games. In contrast, frequency of play showed weak correlations with these variables, suggesting that the perceived learning and motivational value of serious games are not strongly related to digital background or prior gaming literacy (Gee, 2003; Wouters et al., 2013). Methodologically, this association reflects an attitudinal alignment in students’ retrospective appraisals, which may also be influenced by common method variance and individual response-style tendencies.

Non-parametric Spearman rank-order correlations yielded functionally identical coefficients (*rho* = 0.401 to 0.596), confirming the empirical robustness of these associations across ordinal scale assumptions.

### 4.2. Limitations and Future Research

Despite its contributions, this study presents certain limitations that should be acknowledged, along with specific directions for future research to address them.

First, the study relied on a non-probability convenience sampling method, which limits the representativeness of the findings and constrains the extent to which results can be generalized to the broader undergraduate population beyond the participating institutions. Future research should employ probability random sampling methods to compare and validate these findings against those obtained from the current sample.

Second, the data relies on self-reported measures. While self-reporting is appropriate for capturing students’ subjective perceptions, it can introduce potential biases. Self-reported perceived learning cannot be equated with actual learning gains or measured educational effectiveness, it reflects students’ own perceptions of their experience, not an objective outcome. Evaluating cumulative prior experiences rather than a standardized, immediate game intervention introduces recall bias and memory decay. Students’ retrospective appraisals may be subject to salience bias, wherein highly positive, negative, or recent gameplay experiences disproportionately influence overall ratings. Relatedly, because participants were asked to evaluate serious games, in general, rather than a single, common game, the sample of games being appraised was inherently heterogeneous in genre, design quality, and subject matter, which may have introduced additional variability unrelated to the constructs of interest. Future research could overcome this by complementing subjective scales with objective user analytics (learning analytics) or performance-based measures and by standardizing the specific game or game type under evaluation.

Third, this study employs a quantitative approach, which does not capture the underlying reasons behind the observed differences among student profiles. Incorporating qualitative methods in future research would provide a deeper understanding of these factors.

Fourth, although several demographic group differences reached statistical significance, their corresponding effect sizes were predominantly small. This indicates limited practical importance and should not be interpreted as evidence that serious games function similarly or dissimilarly across diverse student groups in any real-world sense. Future research should prioritize effect sizes and could examine these variables through intervention-based designs to determine their true explanatory weight.

Fifth, methodological limitation concerns the potential clustering (nesting) of students within specific classes, academic subjects, and universities. Because recruitment relied on convenience sampling facilitated by course instructors, observations within the same classroom or institution may share unobserved educational contexts and thus may not be entirely independent. Although the analytical strategy prioritized composite-level comparisons, future research should incorporate hierarchical linear modeling (HLM) or cluster-robust standard errors.

Sixth, measuring overall perceived learning and motivation using single-item indicators represents a conceptual and empirical limitation. Consequently, their evidential weight remains exploratory and secondary to the validated multi-item FABLE-S composite dimensions. Also, because all primary variables were collected through self-report questionnaires at a single time point, the observed correlations between perceived learning, motivation, and FABLE-S dimensions may be susceptible to common method variance (CMV) and response-style effects. Future studies should employ multi-wave designs or incorporate objective learning analytics and behavioral logs to isolate cognitive and motivational constructs.

Seventh, the questionnaire was administered in Spanish to the Spanish subsample and in an English-language version to the Dutch subsample. Although the English version was derived from the validated Spanish FABLE-S items, formal measurement equivalence (e.g., cross-language differential item functioning) between the two versions was not established, so linguistic or cultural differences in item interpretation cannot be ruled out as an alternative explanation for the country-related differences reported here.

Eighth, the data used in the present study correspond to the same sample previously employed in the FABLE-S validation study. While this allowed the present analyses to build directly on a psychometrically validated instrument, it also means that the descriptive and group-comparison findings reported here are not based on an independent sample, which may limit the generalizability of the conclusions beyond the characteristics of this particular cohort.

Finally, the cross-sectional nature of this study represents a constraint, as data were collected at a single point in time. This design does not support causal or developmental conclusions about how students’ pedagogical perceptions and motivational levels evolve with continued exposure to serious games. Future longitudinal studies are warranted to track these variables throughout students’ courses and establish the directionality of the relationships identified.

This study addresses the disconnect between traditional higher education pedagogy and the active learning needs of digital-native students by placing the student’s voice at the core of serious games evaluation and responds to the identified research gap by assessing students’ subjective experiences as active users of serious games, rather than focusing on intervention outcomes. By utilizing the FABLE-Scale, the findings provide a key contribution: serious games are perceived as highly engaging and pedagogically promising, but their academic legitimacy depends on stronger alignment with content, instructional practices and curriculum.

## 5. Conclusions

In sum, this research places the student at the center of the study to understand, firsthand, their perception of this educational tool and to evaluate how they value its pedagogical elements, such as content, objectives, and the learning process. The findings show that higher education students highly value the *Learning Process* and *Content and Objectives* dimensions, particularly goal clarity, task scaffolding, and interactivity, while remaining more critical of curriculum integration and the division of academic content into learning units.

Students’ perceptions also varied according to demographics and gaming habits condition. Health and Arts and Humanities students consistently reported the highest valuations across dimensions, whereas Social Sciences students were comparatively most critical. Moreover, frequency of play operates dimensionally, as daily players reported significantly higher scores than never-players. These differences were statistically significant but, in most cases, small in practical terms, and should therefore be interpreted as patterns rather than as evidence of substantially different experiences across groups.

Finally, university students report an overall favorable perception of both the motivation they perceive when playing a serious game and their level of perceived learning. As these are self-reported perceptions rather than objective learning outcomes, they should not be read as evidence of actual educational effectiveness. Among the relationships examined, the association between perceived learning and motivation stood out as the most notable finding, indicating that students who retrospectively rate their learning experience favorably also report higher overall motivation when engaging with serious games. Taken together, these findings indicate that students with prior experience of serious games generally report favorable perceptions of them and underline the value of continuing to investigate their design and implementation from a student-centered perspective.

## Figures and Tables

**Figure 1:**
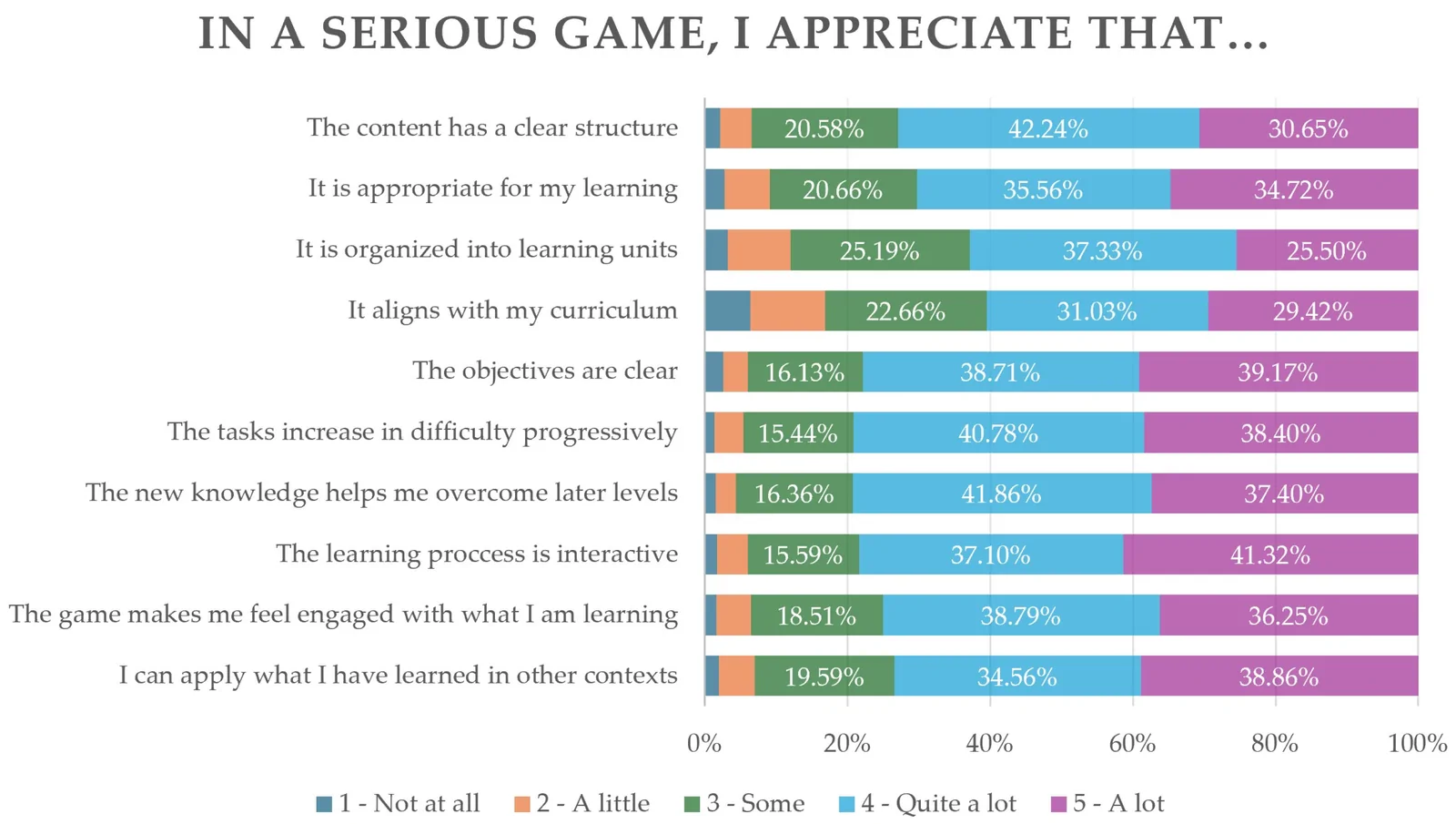
Students’ perception of educational dimensions in serious games.

**Figure 2:**
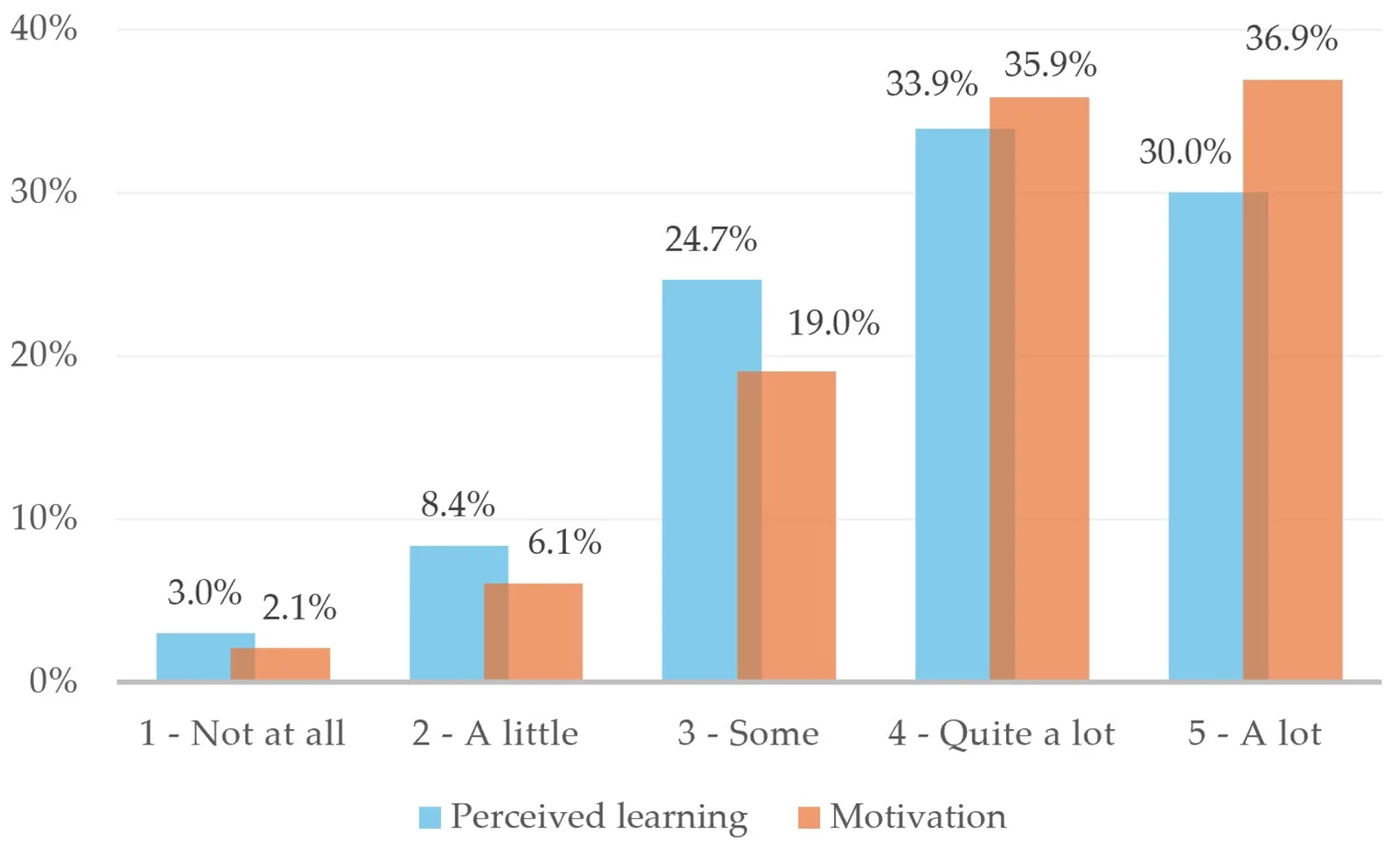
Students’ level of perceived learning and motivation when playing a serious game.

**Table 1:** Sociodemographic and gaming characteristics of the sample.

Variable		*n*	%
Country	Spain	830	63.7
	Netherlands	472	36.3
Age	17–20 y-o	585	44.9
	21–24 y-o	525	40.3
	25 y-o or more	192	14.8
Gender	Male	647	49.7
	Female	610	46.9
	Non-binary	26	2
	Prefer not to say	19	1.4
Academic year	1st year	408	31.3
	2nd year	315	24.2
	3rd year	214	16.5
	4th year	365	28
Area of knowledge	Arts and Humanities	124	9.5
	Social Sciences	390	30
	Sciences	238	18.3
	Health	269	20.6
	Engineering and Architecture	281	21.6
Frequency of play	Never	270	20.7
	Almost never	334	25.7
	Occasionally	354	27.2
	Frequently	231	17.7
	Daily	113	8.7

Note. *Frequency of play* refers to personal gaming habits in daily life, not their frequency of playing serious games in class.

**Table 2:** FABLE-S dimensions.

	Dimension	Description
Ludic experience	*Game structure*	The evaluation of the consistency of the game’s reactions to the player’s actions, the complexity of the controls and the variety of tasks and styles of play, among others.
	*Narrative*	Set of narrative elements of a serious game, such as characters, stories or scenes.
	*Aesthetics*	The measure of the game’s audiovisual quality and its impact on the player’s sensory experience.
	*Interaction*	Keys and degrees of interaction the player has with the game and with other players and the ability to customize and modify the game.
Pedagogical experience	*Content &* *Objective*	The clarity, navigability and appropriateness of the educational content within the serious game, together with the evaluation of its educational goals and their degree of alignment.
	*Learning* *process*	The degree to which the serious game supports a progressive, interactive, and engaging learning process that enables knowledge transfer and application beyond the game context.

Note. Table adapted from the FABLE-S instrument (Merino-Cajaraville & Reyes-de-Cózar, 2026).

**Table 3:** Items from FABLE-S dimensions selected: *Content and Objectives* and *Learning Process*.

Dimension	Items
Content and Objectives	C&O_1—The content has a clear structure. C&O_2—It is appropriate for my learning. C&O_3—It is organized into distinct topics. C&O_4—It aligns with my curriculum. C&O_5—The objectives are clear.
Learning process	PRO_1—The tasks increase in difficulty progressively. PRO_2—The new knowledge helps me overcome later levels. PRO_3—The learning is interactive. PRO_4—The game makes me feel engaged with what I am learning. PRO_5—I can apply what I have learned in other contexts.

Note. Items reproduced from the FABLE-S instrument (Merino-Cajaraville & Reyes-de-Cózar, 2026), corresponding to the two dimensions of the Pedagogical Experience component presented in Table 2.

**Table 4:** Descriptive statistics of *Content and Objectives* dimension items.

Items	*N*	Min.	Max.	M	σ
C&O_1—The content has a clear structure	1302	1.00	5.00	3.95	0.94
C&O_2—It is appropriate for my learning	1302	1.00	5.00	3.93	1.03
C&O_3—It is organized into distinct topics	1302	1.00	5.00	3.73	1.04
C&O_4—It aligns with my curriculum	1302	1.00	5.00	3.67	1.18
C&O_5—The objectives are clear	1302	1.00	5.00	4.08	0.96

**Table 5:** Descriptive statistics of *Learning Process* dimension items.

Items	*N*	Min.	Max.	M	σ
PRO_1—The tasks increase in difficulty progressively	1302	1.00	5.00	4.11	0.90
PRO_2—The new knowledge helps me overcome later levels	1302	1.00	5.00	4.11	0.88
PRO_3—The learning is interactive	1302	1.00	5.00	4.12	0.94
PRO_4—The game makes me feel engaged with what I am learning	1302	1.00	5.00	4.03	0.94
PRO_5—I can apply what I have learned in other contexts	1302	1.00	5.00	4.03	0.98

**Table 6:** Descriptive statistics of *Content and Objectives* and *Learning Process* composite indices.

Items	*N*	Min.	Max.	M	σ
C&O	1302	1.00	5.00	3.87	0.78
PRO	1302	1.00	5.00	4.08	0.72

**Table 7:** Hypothesis testing (ANOVA).

Item/Index	Grouping Variable	Test Statistic	df	*p*_adj	Effect Size	95% CI
C&O	Country	t = −1.59	1043.3	0.678	d = −0.090	[−0.204, 0.023]
C&O	Age	F = 0.48	2534.4	1.000	ω^2^ = 0.000	[0.000, 0.006]
**C&O**	**Gender**	**F = 8.98**	**354.1**	**<0.001**	**ω^2^ = 0.018**	**[0.008, 0.038]**
C&O	Acad. Year	F = 0.17	3655.5	1.000	ω^2^ = 0.000	[0.000, 0.006]
C&O	Area know.	F = 3.51	4534.0	0.061	ω^2^ = 0.007	[0.000, 0.022]
C&O	Freq. play	F = 2.53	4505.6	0.277	ω^2^ = 0.004	[0.000, 0.019]
C&O_1	Country	t = −1.13	1004.1	0.394	d = −0.065	[−0.178, 0.048]
C&O_1	Age	F = 0.17	2534.0	0.934	ω^2^ = 0.000	[0.000, 0.005]
**C&O_1**	**Gender**	**F = 7.42**	**353.6**	**0.002**	**ω^2^ = 0.015**	**[0.004, 0.035]**
C&O_1	Acad. Year	F = 0.19	3656.6	0.964	ω^2^ = 0.000	[0.000, 0.006]
C&O_1	Area know.	F = 1.99	4535.6	0.228	ω^2^ = 0.002	[0.000, 0.015]
**C&O_1**	**Freq. play**	**F = 4.04**	**4513.2**	**0.013**	**ω^2^ = 0.007**	**[0.001, 0.023]**
C&O_2	Country	t = −1.43	994.2	0.317	d = −0.082	[−0.195, 0.031]
C&O_2	Age	F = 1.68	2536.7	0.340	ω^2^ = 0.001	[0.000, 0.010]
**C&O_2**	**Gender**	**F = 5.69**	**353.4**	**0.009**	**ω^2^ = 0.011**	**[0.003, 0.028]**
C&O_2	Acad. Year	F = 1.22	3655.2	0.439	ω^2^ = 0.001	[0.000, 0.012]
**C&O_2**	**Area know.**	**F = 6.59**	**4532.8**	**<0.001**	**ω^2^ = 0.016**	**[0.006, 0.035]**
C&O_2	Freq. play	F = 1.65	4501.5	0.322	ω^2^ = 0.002	[0.000, 0.016]
C&O_3	Country	t = −1.17	1007.9	0.394	d = −0.067	[−0.180, 0.046]
C&O_3	Age	F = 0.19	2533.5	0.934	ω^2^ = 0.000	[0.000, 0.005]
**C&O_3**	**Gender**	**F = 5.74**	**353.2**	**0.009**	**ω^2^ = 0.011**	**[0.003, 0.028]**
C&O_3	Acad. Year	F = 1.34	3649.7	0.394	ω^2^ = 0.001	[0.000, 0.012]
C&O_3	Area know.	F = 2.14	4524.8	0.194	ω^2^ = 0.003	[0.000, 0.017]
C&O_3	Freq. play	F = 1.61	4495.9	0.331	ω^2^ = 0.002	[0.000, 0.016]
C&O_4	Country	t = −2.08	1047.9	0.111	d = −0.118	[−0.231, −0.005]
C&O_4	Age	F = 0.06	2536.5	0.974	ω^2^ = 0.000	[0.000, 0.004]
C&O_4	Gender	F = 3.25	353.6	0.091	ω^2^ = 0.005	[0.000, 0.018]
C&O_4	Acad. Year	F = 0.19	3655.5	0.964	ω^2^ = 0.000	[0.000, 0.006]
C&O_4	Area know.	F = 1.85	4525.4	0.264	ω^2^ = 0.002	[0.000, 0.016]
C&O_4	Freq. play	F = 1.39	4500.1	0.394	ω^2^ = 0.001	[0.000, 0.014]
C&O_5	Country	t = 0.05	965.0	0.974	d = 0.003	[−0.110, 0.116]
C&O_5	Age	F = 0.30	2548.6	0.892	ω^2^ = 0.000	[0.000, 0.005]
**C&O_5**	**Gender**	**F = 10.54**	**355.8**	**<0.001**	**ω^2^ = 0.017**	**[0.007, 0.033]**
C&O_5	Acad. Year	F = 0.46	3652.8	0.891	ω^2^ = 0.000	[0.000, 0.008]
C&O_5	Area know.	F = 1.49	4527.5	0.360	ω^2^ = 0.001	[0.000, 0.013]
C&O_5	Freq. play	F = 2.55	4513.7	0.111	ω^2^ = 0.003	[0.000, 0.017]
**PRO**	**Country**	**t = 2.89**	**977.4**	**0.035**	**d = 0.167**	**[0.054, 0.280]**
PRO	Age	F = 0.14	2525.4	1.000	ω^2^ = 0.000	[0.000, 0.004]
PRO	Gender	F = 0.65	354.3	1.000	ω^2^ = 0.000	[0.000, 0.009]
PRO	Acad. Year	F = 1.48	3644.2	1.000	ω^2^ = 0.001	[0.000, 0.013]
**PRO**	**Area know.**	**F = 4.81**	**4540.8**	**0.008**	**ω^2^ = 0.013**	**[0.004, 0.032]**
**PRO**	**Freq. play**	**F = 9.76**	**4520.3**	**<0.001**	**ω^2^ = 0.023**	**[0.011, 0.046]**
PRO_1	Country	t = 1.32	921.4	0.340	d = 0.077	[−0.036, 0.190]
PRO_1	Age	F = 0.37	2528.3	0.884	ω^2^ = 0.000	[0.000, 0.006]
PRO_1	Gender	F = 2.33	353.2	0.212	ω^2^ = 0.003	[0.000, 0.017]
PRO_1	Acad. Year	F = 0.43	3651.2	0.892	ω^2^ = 0.000	[0.000, 0.008]
**PRO_1**	**Area know.**	**F = 3.14**	**4530.3**	**0.048**	**ω^2^ = 0.008**	**[0.001, 0.026]**
**PRO_1**	**Freq. play**	**F = 8.22**	**4512.9**	**<0.001**	**ω^2^ = 0.023**	**[0.009, 0.045]**
**PRO_2**	**Country**	**t = 4.91**	**895.1**	**<0.001**	**d = 0.287**	**[0.174, 0.401]**
PRO_2	Age	F = 0.22	2538.6	0.934	ω^2^ = 0.000	[0.000, 0.005]
PRO_2	Gender	F = 1.07	353.2	0.522	ω^2^ = 0.001	[0.000, 0.015]
PRO_2	Acad. Year	F = 1.97	3645.0	0.264	ω^2^ = 0.002	[0.000, 0.015]
**PRO_2**	**Area know.**	**F = 3.52**	**4530.0**	**0.029**	**ω^2^ = 0.009**	**[0.001, 0.027]**
**PRO_2**	**Freq. play**	**F = 8.64**	**4518.0**	**<0.001**	**ω^2^ = 0.022**	**[0.010, 0.043]**
**PRO_3**	**Country**	**t = 3.09**	**876.8**	**0.010**	**d = 0.181**	**[0.068, 0.294]**
PRO_3	Age	F = 0.52	2520.6	0.796	ω^2^ = 0.000	[0.000, 0.007]
PRO_3	Gender	F = 0.32	353.6	0.934	ω^2^ = 0.000	[0.000, 0.007]
PRO_3	Acad. Year	F = 1.33	3642.5	0.394	ω^2^ = 0.001	[0.000, 0.013]
**PRO_3**	**Area know.**	**F = 3.98**	**4533.8**	**0.014**	**ω^2^ = 0.009**	**[0.002, 0.027]**
**PRO_3**	**Freq. play**	**F = 5.67**	**4508.0**	**0.002**	**ω^2^ = 0.014**	**[0.005, 0.034]**
PRO_4	Country	t = −1.49	1041.1	0.294	d = −0.085	[−0.198, 0.028]
PRO_4	Age	F = 0.00	2542.8	0.999	ω^2^ = 0.000	[0.000, 0.004]
PRO_4	Gender	F = 0.59	354.0	0.816	ω^2^ = 0.000	[0.000, 0.006]
PRO_4	Acad. Year	F = 0.92	3645.5	0.584	ω^2^ = 0.000	[0.000, 0.010]
PRO_4	Area know.	F = 2.22	4541.1	0.179	ω^2^ = 0.003	[0.000, 0.017]
**PRO_4**	**Freq. play**	**F = 4.94**	**4514.8**	**0.004**	**ω^2^ = 0.009**	**[0.002, 0.026]**
**PRO_5**	**Country**	**t = 3.19**	**901.7**	**0.009**	**d = 0.186**	**[0.073, 0.300]**
PRO_5	Age	F = 0.09	2529.2	0.964	ω^2^ = 0.000	[0.000, 0.004]
PRO_5	Gender	F = 1.41	353.0	0.394	ω^2^ = 0.002	[0.000, 0.019]
PRO_5	Acad. Year	F = 1.04	3645.5	0.522	ω^2^ = 0.000	[0.000, 0.011]
**PRO_5**	**Area know.**	**F = 6.61**	**4532.4**	**<0.001**	**ω^2^ = 0.017**	**[0.006, 0.036]**
**PRO_5**	**Freq. play**	**F = 3.23**	**4505.6**	**0.044**	**ω^2^ = 0.007**	**[0.001, 0.022]**

Note. *p* values were corrected for multiple testing separately within each family: Holm–Bonferroni for the primary, composite-level tests (*n* = 12) and Benjamini–Hochberg false discovery rate (FDR) for the exploratory, item-level tests (*n* = 60); *p*_adj denotes the corrected value. Cohen’s d is reported for the two-group comparison (Country) and omega-squared (ω^2^) for the omnibus (>2-group) comparisons; as these effect sizes are not directly comparable in magnitude, pairwise d values were not computed for the latter to avoid additional multiplicity correction. The pairs highlighted in bold are those that show statistically significant differences.

**Table 8:** Post hoc test using Games–Howell.

	Variable	*F*/*t*	*p*	Post Hoc Test	M	M Diff.	*p*-Value	*d* Cohen
C&O	Gender	8.98	<0.001	Female–Male	3.99–3.76	0.228	<0.001	0.295
PRO	Country	2.89	0.035	Spain–Netherlands	4.12–4.00	0.121	0.004	0.167
	Area knowledge	4.81	0.008	Engineering and Architecture–Social Sciences	4.15–3.96	0.188	0.012	0.261
				Arts and Humanities–Social Sciences	4.23–3.96	0.268	0.001	0.372
				Health–Social Sciences	4.14–3.96	0.176	0.033	0.244
	Frequency of play	9.76	<0.001	Daily-Never	4.34–3.92	0.421	<0.001	0.592
				Daily-Almost never	4.34–4.02	0.324	<0.001	0.449
				Daily-Occasionally	4.34–4.13	0.209	0.006	0.296
				Daily-Frequently	4.34–4.14	0.197	0.022	0.275
				Occasionally-Never	4.13–3.92	0.212	0.009	0.296
				Frequently-Never	4.14–3.92	0.227	0.008	0.317

**Table 9:** Correlation between educational dimensions, perceived learning, motivation, and frequency of play.

		C&O	PRO	Perceived Learning	Motivation
Perceived learning	Pearson’s *r*	0.484	0.477	1	
	*p*-value	<0.001	<0.001	-	
	Spearman’s *rho*	0.453	0.457	1	
	*p*-value	<0.001	<0.001	-	
Motivation	Pearson’s *r*	0.425	0.432	0.589	1
	*p*-value	<0.001	<0.001	<0.001	-
	Spearman’s *rho*	0.401	0.401	0.596	1
	*p*-value	<0.001	<0.001	<0.001	-
Frequency of play	Pearson’s *r*	0.042	0.070	0.020	0.156
	*p*-value	0.133	0.012	0.468	<0.001
	Spearman’s *rho*	−0.004	0.064	0.027	0.123
	*p*-value	0.879	0.020	0.338	<0.001

## Data Availability

The data presented in this study are openly available in the Zenodo repository at the following permanent link: https://doi.org/10.5281/zenodo.20338078.

## Note

1 Regarding the gender variable, while all four self-reported categories (Male, Female, Non-binary, and Prefer not to say) were incorporated into the Welch’s ANOVA due to its resilience under sample size imbalance, pairwise Games–Howell post hoc evaluations were restricted to Female and Male comparisons. Non-binary (*n* = 26, 2.0%) and prefer not to say (*n* = 19, 1.4%) subgroups were excluded from post hoc pairwise testing due to insufficient statistical power.

## Supplementary Materials

The following supporting information can be downloaded at: https://www.mdpi.com/article/10.3390/bs16101766/s1, Table S1: Sample distribution by country and participating university.
