# Supplementary material for: Fun and Balanced Learning Experience: Students’ Perceptions of Serious Games in Higher Education
Source: Behav Sci (Basel). 2026 Sep 28;16(10):1766. doi: 10.3390/bs16101766 (PMC13701390; doi:10.3390/bs16101766)
Supplement: Supplementary file 1 [file behavsci-16-01766-s001.zip › behavsci-4473808-supplementary.pdf]

Table S1. Sample distribution by country and participating university.

**Country**	**University/Institution**	*n*	%
**Spain**		**830**	**63.75%**
	Universidad Loyola	830	63.75%
**Netherlands**		**472**	**36.25%**
	University of Amsterdam	80	6.14%
	Hogeschool	77	5.91%
	Eindhoven University of Technology	76	5.84%
	Breda University of Applied Sciences	75	5.76%
	Erasmus University Rotterdam	62	4.76%
	Other	30	2.30%
	TU Delft	22	1.69%
	Utrecht University	17	1.31%
	Vrije University Amsterdam	17	1.31%
	Maastricht University	9	0.69%
	Tilburg University	7	0.54%
**Total**		**1302**	**100 %**
